# Nutrient Management on Soil Microbiome Dynamics and Plant Health

**DOI:** 10.3390/plants15182830

**Published:** 2026-09-16

**Authors:** Chenliang Yu

**Affiliations:** National Key Laboratory for Development and Utilization of Forest Food Resources, Zhejiang A&F University, Hangzhou 311300, China; yuchenliang@zafu.edu.cn

## 1. Introduction

Soils harbor the most diverse microbial communities on Earth. A single gram of soil can contain billions of microorganisms spanning thousands of taxa, and these communities form the biological engine that drives nutrient cycling, organic matter decomposition, and plant–microbe interactions. Far from being passive recipients of mineral nutrients, plants are intimately connected to this invisible network through their roots, which shape, and are in turn shaped by, the surrounding microbiome. Understanding how nutrient management modulates this relationship has therefore become a central question at the interface of agricultural science, ecology, and soil biology.

Nutrient management—the deliberate regulation of the supply of essential elements such as carbon, nitrogen, phosphorus, potassium, and micronutrients—exerts a profound and often underestimated influence on soil microbial community dynamics and, through them, on plant health. From a scientific perspective, nutrient supply directly modulates microbial metabolic pathways and community structure, potentially favoring beneficial microorganisms, depending on nutrient rates, soil conditions, and the resident community. From an applied perspective, well-designed fertilization strategies—including organic fertilizers, biofertilizers, and balanced mineral fertilizer formulations—have been widely adopted to improve soil fertility, reduce pest and disease incidence, and enhance crop resistance to abiotic stresses such as drought and salinity. These bottom-up regulatory mechanisms hold the key to the green transition and sustainable development of global agriculture.

The Special Issue “Nutrient Management on Soil Microbiome Dynamics and Plant Health” was launched to bring together the latest research on this topic. It has successfully gathered 11 contributions—nine research articles and two review articles—from authors across Asia and Europe. Collectively, these papers span a remarkable diversity of agricultural and forestry systems, including tree crops (*Torreya grandis*, *Camellia oleifera*), horticultural crops (strawberry, goji berry, tea), staple crops (maize, potato), and subtropical forests (*Moso bamboo*), and investigate nutrient management strategies ranging from phosphate-solubilizing microbial inoculation and organic–biological co-amendment to nitrogen–zinc co-fertilization, silicon-based soil conditioning, and phosphorus tailing recycling. The breadth of this coverage reflects the growing recognition that sustainable nutrient management must be understood, and designed, at the level of the soil–plant–microbe continuum.

## 2. Overview of the Special Issue Contributions

The contributions to this Special Issue can be broadly organized into four complementary themes that together chart the current landscape of nutrient-driven microbiome research. The first theme addresses microbiome-driven nutrient mobilization and soil fertility, examining how beneficial microorganisms—either introduced or managed—activate recalcitrant nutrient pools. The second theme concerns fertilization regimes and their capacity to reshape rhizosphere microbiomes and, consequently, plant health. The third theme explores microbiome-mediated disease suppression and plant resilience, highlighting the protective functions of the soil microbiome against soil-borne pathogens and abiotic stress. The fourth theme extends the scope upward, from molecular nutrient signaling within plants to ecosystem-level carbon cycling, illustrating how nutrient management connects to global sustainability goals.

Li et al. [1] demonstrated that inoculating *Torreya grandis* seedlings with a phosphate-solubilizing *Burkholderia* strain significantly increased soil total phosphorus, potassium, and nitrogen concentrations, markedly enhanced available phosphorus, and reshaped both bacterial (*Acidobacteria*, *Chloroflexi*, *Proteobacteria*) and fungal (*Ascomycota*) communities, with metabolomic analyses revealing coordinated shifts in fatty acids, hormones, and amino acids that linked key microbial taxa to soil phosphorus metabolism. Complementing this empirical work, Zhu et al. [2] provided a comprehensive review of phosphate-solubilizing microbial interactions in plant–soil systems, synthesizing mechanisms of phosphorus activation and advocating for the use of microbial interactions and synthetic communities as an entry point to engineer functional rhizosphere microbiomes. Geng et al. [3] reported that two consecutive applications of phosphate tailings (PTs) at 9 t·ha^−1^ in an acidic red-soil corn–potato rotation increased late-autumn potato yield by 12.82% and improved soil quality indices in the potato cropping seasons, and elevated soil pH by 0.72–1.58 units, while enriching bacterial groups potentially involved in phosphorus mobilization (Actinomycetota, Chloroflexota) and altering bacterial–fungal co-occurrence networks—suggesting complementary roles of acid–base balance, structural improvement, and microbial activation. Zhu et al. [4] further showed that mixing *Moso bamboo* with *Liquidambar formosana* created a balanced soil nutrient profile and enriched carbon-decomposing taxa (*Terriglobales*, *Sphingomonas*), whereas mixing with Phoebe chekiangensis favored acid-tolerant nitrogen-cycling groups, indicating that species-specific mixed planting can sustainably modify soil nutrient status and microbial function. Jakutis et al. [5] showed that excessive mineral nitrogen reduced microbial abundance and disrupted soil nitrogen cycling, whereas organic and biological amendments mitigated these effects. They recommended limiting nitrogen inputs to 150 kg N·ha^−1^, or 180 kg N·ha^−1^ with organic fertilizer. Lu et al. [6] showed that nitrogen–zinc co-fertilization in tea (*Camellia sinensis*) rhizosphere soil increased total nitrogen and mineral nitrogen availability, recruited rhizobacteria associated with nitrogen cycling and zinc activation (*Proteobacteria*, *Acidobacteriota*, *Gemmatimonadota*), and interfered with lipid, amino acid, and cofactor–vitamin metabolism, with soil pH and total nitrogen identified as dominant drivers of both bacterial community and metabolite shifts. Sun et al. [7] demonstrated that the co-application of soluble silicon and activated carbon markedly enhanced strawberry salt tolerance by reinforcing ion–redox homeostasis—increasing shoot and root fresh weight by 67.5% and 78.5%, reducing shoot Na+ by 59.1%, and lowering H_2_O_2_ and malondialdehyde by 62.6% and 66.5%—while activated carbon concurrently alleviated salt-induced increases in soil electrical conductivity, improved nutrient availability, and restructured the rhizosphere bacterial community. Wang et al. [8] linked goji berry root rot to rhizosphere fungal dysbiosis characterized by Fusarium enrichment and reduced network stability. Native antagonistic Trichoderma strains were identified as promising agents for microbiome-based disease control. Chen et al. [9] found that combining validamycin A with *Bacillus velezensis* TCS001 enhanced the control of Camellia anthracnose by directly inhibiting the pathogen and activating host defenses, demonstrating the potential of integrated chemical–biological control. Chen et al. [10] reviewed the NRT1.1–NLP7 nitrate-signaling nexus and proposed a structure-guided framework for improving nitrogen-use efficiency. At the landscape scale, Xu et al. [11] identified Zhejiang Province as a strengthening vegetation carbon sink shaped by interacting natural and anthropogenic factors, emphasizing the need for coordinated ecosystem management.

## 3. Key Findings and Thematic Synthesis

### 3.1. Microbiome-Driven Nutrient Mobilization and Soil Fertility

A central message emerging from this Special Issue is that the microbial community is not merely a passive responder to fertilization but an active agent of nutrient mobilization. Whether through the inoculation of phosphate-solubilizing bacteria in *Torreya grandis* [1], the theoretical framework of phosphate-solubilizing microbial interactions and synthetic communities [2], the recycling of phosphorus tailings into acidic agricultural soils [3], or the design of species-specific mixed plantings in bamboo forests [4], the papers consistently show that microbial community structure and function are the important intermediaries linking nutrient input to plant performance. Notably, the phosphorus tailing study [3] demonstrates that phosphate tailings may serve as useful soil amendments when applied at appropriate rates when they are leveraged to activate indigenous microbial functions, providing a compelling example of circular economy principles applied to soil fertility management.

### 3.2. Fertilization Regimes Reshaping Rhizosphere Microbiomes and Plant Health

The second key finding concerns the dual nature of fertilization. Mineral nitrogen, while essential for crop productivity, can destabilize soil microbial communities when applied in excess, as shown by reduced total microbial abundance, lowered fungal-to-bacterial ratios, and diminished biological nitrogen immobilization in intensive cropping systems [5]. Importantly, the negative microbial effects of mineral nitrogen can be mitigated through co-application with organic fertilizers and biological activators, which restore fungal abundance and improve nitrogen transformation efficiency. Similar principles apply to micronutrient management: nitrogen–zinc co-fertilization in tea improved nitrogen availability and recruited beneficial rhizobacteria with minimal disturbance to microbial diversity [6], while silicon combined with activated carbon simultaneously relieved ionic and oxidative stress in strawberry and reconditioned the rhizosphere microbiome [7]. These findings collectively support a paradigm shift from single-nutrient, yield-only fertilization toward coordinated, microbiome-conscious nutrient management.

### 3.3. Microbiome-Mediated Disease Suppression and Plant Resilience

The Special Issue also underscores the protective dimension of the soil microbiome. The goji berry root rot study [8] provides compelling evidence that disease outbreaks are rooted in a loss of microbial network stability—a shift from a complex, disease-suppressive community enriched in *Trichoderma* to a simplified, pathogen-dominated community enriched in *Fusarium*—rather than in simple species loss. This ecological perspective suggests that disease management should consider the recovery of microbial community stability in addition to pathogen suppression. Similarly, the combination of validamycin A with Bacillus velezensis TCS001 [9] illustrates how biocontrol agents can be integrated with reduced chemical inputs to achieve synergistic disease suppression while activating host defenses. Together, these studies position the management of microbial community structure—through biocontrol inoculation, organic matter inputs, and diversified cropping—as a cornerstone of sustainable disease management.

### 3.4. From Nutrient Signaling to Ecosystem Function

Finally, the Special Issue connects soil microbiome research to broader biological and environmental scales. At the molecular level, the review of the NRT1.1–NLP7 nexus [10] reveals how nitrate, the most important mineral nutrient input in agriculture, is perceived and transduced as both a nutrient and a signal, and how structure-guided engineering of this nexus could improve nitrogen-use efficiency—a goal of central importance for reducing fertilizer waste and environmental pollution. At the landscape scale, the Zhejiang carbon sink study [11] demonstrates how vegetation management and ecological restoration, which depend fundamentally on soil fertility and plant health, contribute to regional carbon sequestration. Together, these studies place nutrient management within a broader framework extending from plant signaling and microbial processes to ecosystem function.

## 4. Recent Advances, Research Gaps, and Future Directions

Collectively, the studies in this Special Issue reflect a transition from descriptive microbiome surveys toward more predictive and mechanism-based nutrient management. Recent methodological advances, including the integration of amplicon sequencing with untargeted metabolomics [1,6], transcriptomic analysis of pathogen responses [9], microbial co-occurrence network analysis [3,8], and remote sensing and geodetector-based ecosystem modeling [11], have improved our understanding of the relationships among microbial taxa, metabolic functions, plant phenotypes, and ecosystem processes. Meanwhile, the development of phosphate-solubilizing microbial consortia [2], microbiome-oriented disease control strategies [8,9], and structure-guided engineering of the NRT1.1–NLP7 nitrate signaling nexus [10] reflects growing interest in the rational manipulation of plant–soil–microbe systems. Nevertheless, many reported relationships remain correlational, requiring causal experiments and broader field validation [5,6]. Long-term monitoring is also needed to assess the environmental safety of recycled amendments [3], while standardized methods are essential for translating beneficial microorganisms into reliable products [2,8,9]. Future research should prioritize microbiome-informed fertilization [1,2,3,4,5], engineering of plant nutrient signaling [10], robust synthetic microbial communities [2,8,9], and integrated nutrient-management systems combining mineral, organic, biological, and recycled inputs [3,5]. Finally, high-resolution remote sensing, long-term ecological monitoring, and predictive modeling will be essential for scaling rhizosphere-level knowledge to ecosystem services, carbon sequestration, and regional food-security outcomes [11]. Achieving these goals will require interdisciplinary collaboration among microbiologists, plant scientists, soil scientists, agronomists, ecologists, breeders, and data scientists.

## Data Availability

No new data were created or analyzed in this study. Data sharing is not applicable to this article.
